# Cla4p Kinase Activity Is Down-Regulated by Fus3p during Yeast Mating

**DOI:** 10.3390/biom12040598

**Published:** 2022-04-18

**Authors:** Junwon Kim, Mark D. Rose

**Affiliations:** 1 Department of Molecular Biology, Princeton University, Princeton, NJ 08544, USA; junwon.kim@georgetown.edu; 2 Department of Biology, Georgetown University, Washington, DC 20057, USA

**Keywords:** PAK-kinase, MAP-kinase, *Saccharomyces cerevisiae*, conjugation, budding yeast, phosphorylation, nuclear localization

## Abstract

In *Saccharomyces cerevisiae*, the p21-activated kinase Cla4p regulates polarized morphogenesis and cytokinesis. However, it remains unknown how Cla4p kinase activity is regulated. After pheromone exposure, yeast cells temporally separate the mitotic and mating programs by sequestering Fus2p in the nucleus until cell cycle completion, after which Fus2p exits to facilitate cell fusion. Previously, we showed that sequestration is regulated by two opposing protein kinases, Cla4p and Fus3p. Phosphorylation of Fus2p-S67 by Cla4p promotes nuclear localization by both activating nuclear import and blocking export. During mating, phosphorylation of Fus2p-S85 and Fus2p-S100 by Fus3p promotes nuclear export and blocks import. Here, we find that Cla4p kinase activity is itself down-regulated during mating. Pheromone exposure causes Cla4p hyper-phosphorylation and reduced Fus2p-S67 phosphorylation, dependent on Fus3p. Multiple phosphorylation sites in Cla4p are mating- and/or Fus3p-specific. Of these, Cla4p-S186 phosphorylation reduced the kinase activity of Cla4p, in vitro. A phosphomimetic *cla4-S186E* mutation caused a strong reduction in Fus2p-S67 phosphorylation and nuclear localization, in vivo. More generally, a non-phosphorylatable mutation, *cla4-S186A*, caused failure to maintain pheromone arrest and delayed formation of the mating-specific septin morphology. Thus, as cells enter the mating pathway, Fus3p counteracts Cla4p kinase activity to allow proper mating differentiation.

## 1. Introduction

The Rho family of small GTPases comprises key molecular switches that control a wide range of signal transduction pathways. Among other outputs, the Rho-proteins control cell morphology in eukaryotic cells through the reorganization of the cytoskeleton in response to extracellular stimuli. In budding yeast, the Rho family GTPase Cdc42p physically interacts with and activates Ste20p and Cla4p, two members of a highly conserved family of p21-activated kinases (PAKs). Ste20p and Cla4p have distinct roles, but share at least one essential function [1]. Ste20p acts as an upstream activator of multiple mitogen-activated protein kinase (MAPK) cascades that regulate different types of polarized growth including mating, filamentous growth, and osmotolerance [2,3,4].

In contrast, Cla4p, a homolog of Ste20p, functions during vegetative cell growth. Cla4p was initially identified in a screen for proteins required for viability in the absence of the G1 cyclins, Cln1p and Cln2p [5,6]. After shut-off of *CLN* gene expression, *cla4* mutants arrest with wide mother-bud necks, elongated buds, and multiple nuclei, indicating a functional connection with bud neck formation, polarized growth, and cytokinesis. Several proteins regulating cell polarization have been identified as downstream targets for Cla4p. Two myosin I homologues, Myo3p and Myo5p, are phosphorylated, in vitro, by Cla4p and Ste20p at sites that are required for polarization of the actin cytoskeleton, in vivo [7,8]. Cla4p is recruited to the bud tip by GTP-Cdc42p and PI4P [9]. Cla4p and Ste20p recruit the scaffolding protein, Bem1p, forming a complex with and phosphorylating Cdc24p, the guanine nucleotide exchange factor for Cdc42p [10,11], to regulate polarized bud growth [12,13]. The best characterized function of Cla4p is the regulation of septin structure, consistent with Cla4p involvement in bud morphogenesis and cytokinesis [14,15,16,17,18]. Cla4p directly phosphorylates at least two septins, Cdc3p and Cdc10p; phosphorylation is essential for septin filament assembly and collar formation during bud emergence and cytokinesis. In addition, cell cycle progression is partially regulated by Cla4p. Along with polo kinase Cdc5p, Cla4p acts in the stepwise phosphorylation and down-regulation of Swe1p, an inhibitor of entry into mitosis [19]. Cla4p also contributes to mitotic exit by phosphorylating Lte1p, which promotes Lte1p’s localization at the bud cell cortex to trigger exit from mitosis [20,21,22].

Cla4p kinase activity is cell-cycle-dependent. In vitro assay of Cla4p kinase activity from protein immunoprecipitated from cell extracts shows high levels during mitosis, which drops as cells complete cytokinesis and enter G1 [5]. The kinase activity of Cla4p is stimulated by binding the GTP-bound form of Cdc42p, which alleviates autoinhibition of the catalytic domain by Cla4p’s Cdc42p binding domain [5]. Cla4p is hyper-phosphorylated during mitosis, dependent on CDK/Clb2p and GTP-bound Cdc42p [23]; hyper-phosphorylation of Cla4p is required in vivo for the phosphorylation and activation of Gin4p, a protein kinase responsible for septin reorganization during mitosis. However, mitotic hyper-phosphorylation had no significant effects on in vitro Cla4p kinase activity [23]. Thus, it still remains unclear how mitotic CDK and phosphorylation regulates Cla4p.

Previously, we identified Fus2p as a novel target for Cla4p. Fus2p is a key regulator of cell fusion during mating; phosphorylation of Fus2p by Cla4p is part of the mechanism that keeps the mitotic and mating programs temporally separate [24]. Even before cells complete mitosis, Fus2p is highly induced by pheromone; however, it is sequestered in the nucleus until cells complete cytokinesis [25,26]. Only after mitosis, as cells arrest in G1, does Fus2p exit the nucleus. In the cytoplasm, Fus2p co-opts mitotic proteins (Cdc42p and Rvs161p) from different functions to regulate cell wall removal at the zone of cell fusion [27,28,29]. Fus2p’s nuclear localization is regulated in opposing ways by phosphorylation. A nuclear export signal (NES) in Fus2p is activated by phosphorylation of S84 and S100 by the pheromone-activated MAP-kinase Fus3p [24,26]. Although active Fus3p begins to phosphorylate Fus2p prior to cytokinesis, during mitosis, Cla4p continues to promote the nuclear localization of Fus2p by phosphorylating Fus2p-S67. Phosphorylation of S67 both activates the bipartite nuclear localization signal (NLS) and inactivates the NES by reducing the interaction with an exportin, Crm1p [24]. 

Fus2p-S67 phosphorylation is epistatic to activation of the NES by Fus3p [24]. This implies that Cla4p’s kinase activity must be down-regulated during mating to allow Fus2p to exit the nucleus. In this study, we examined Fus2p localization and phosphorylation during the transition between mitosis and mating to understand how Cla4p kinase activity is regulated. As cells exit mitosis and begin to differentiate into mating cells, Fus2p-S67 phosphorylation is down-regulated as Cla4p becomes hyper-phosphorylated. Mutant screens and biochemical studies revealed that Fus3p negatively regulates Cla4p kinase activity by inhibitory phosphorylation of Cla4p-S186. As a consequence, Fus2p-S67 phosphorylation is blocked, thereby allowing Fus2p to remain cytoplasmic during mating. This is the first demonstration of a regulatory mechanism for this important PAK kinase.

## 2. Materials and Methods

### 2.1. Yeast Strains, Plasmids, and General Methods

All yeast culture and genetic techniques were performed as previously described [24]. *S. cerevisiae* strains used in this study are listed in Table S1 in the Supplementary Materials. Yeast strains were constructed using a PCR-based one-step gene disruption technique [30]. Various deletion constructs were constructed on centromeric shuttle plasmids and transformed in to yeast for analysis [31]. The *GAL1* promoter was induced for full-length Fus2p and Cla4p expression using 2% galactose and for Fus2p truncations with 2% galactose + 0.2% glucose. To induce mating differentiation, 10 µg/mL α-factor was used. Plasmids used in this study are listed in Table S2 in the Supplementary Materials. Point mutations of *FUS2* or *CLA4* were generated by PCR-based site-directed mutagenesis. Proteins were prepared by TCA precipitation, resolved on gels with or without Phos-tag (Wako Chemicals, Richmond, VA, USA), and detected on Western blots with monoclonal anti-GFP (Roche, South San Francisco, CA, USA), monoclonal anti-c-Myc (Sigma, St. Louis, MO, USA), monoclonal-HA (Thermo Scientific, Waltham, MA, USA) and monoclonal anti-MBP (Millipore, Burlington, MA USA). Band intensity was quantified using a G:BOX imaging system (Syngene, Frederick, MD, USA).

### 2.2. Microscopy and Imaging Analysis

Fluorescence microscopy and quantification of GFP florescence intensity were performed as described previously [24,26]. Images were visualized on a microscopy system (DeltaVision; Applied Precision, LLC, Issaquah, WA, USA) using an inverted microscope (TE200; Nikon Instruments, Melville, NY, USA), a charge-coupled device camera (CoolSNAP HQ; Roper Scientific, Tucson, AZ, USA) and either a 40× objective with a 0.75 NA or a 100× objective with a 1.4 NA (Nikon). For time-lapse microscopy, cells were grown to early exponential phase at 30 °C and treated with α-factor for 90 min. At t = 0, either 100 µM of 1-NA-PP1 or an equal volume of DMSO was added. Image stacks were acquired every 1 min. Image stacks were deconvolved and flattened using softWoRx (Applied Precision, LLC). For the ratio of cytoplasmic to nuclear fluorescence, the intensity of selected regions in the nucleus or cytoplasm was quantified by softWoRx or Image J (NIH). Background fluorescence was subtracted by placing the same measurement circle in nearby regions without a GFP signal.

### 2.3. Screening for Pheromone and Fus3p-Specific Phosphorylation Sites of Cla4p

The Cla4p truncations are listed in Table S2 in the Supplementary Materials. All were fused C-terminally to triple GFP (GPF_3_) and expressed under the control of the *GAL1* promoter for 2 h in actively growing cells or cells G1-arrested by α-factor for 90 min. Each truncation was transformed into *FUS3* (MY11198) and *fus3*Δ *cln3*Δ (MY10273) strains, both of which carry *cdc28-as1*. To investigate CDK-dependence, *cdc28-as1* cells were treated with 500 nM of 1-NM-PP1 for 30 min before Cla4p induction by 2% galactose. 

### 2.4. In Vitro Cla4p Kinase Assay

HA-tagged wild-type or mutant Cla4p were immune-precipitated by incubating total cell lysate at 4 °C for 2 h with anti-HA magnetic beads (Pierce). For the in vitro kinase assay, purified Cla4p was mixed with 1 µg of dephosphorylated MBP (Millipore) in 15 μL kinase buffer (50 mM Tris-Cl, pH 7.5, 10 mM MgCl_2_, 1 mM DTT) and 200 mM ATP. After incubation at 30 °C for 1 h, SDS sample buffer was added to terminate the phosphorylation reaction. Proteins were separated by 7.5% SDS-PAGE containing 50 µM Phos-tag and detected by anti-MBP.

## 3. Results

### 3.1. Fus3p Down-Regulates Fus2p-S67 Phosphorylation by Cla4p during Mating 

**Figure 1:**
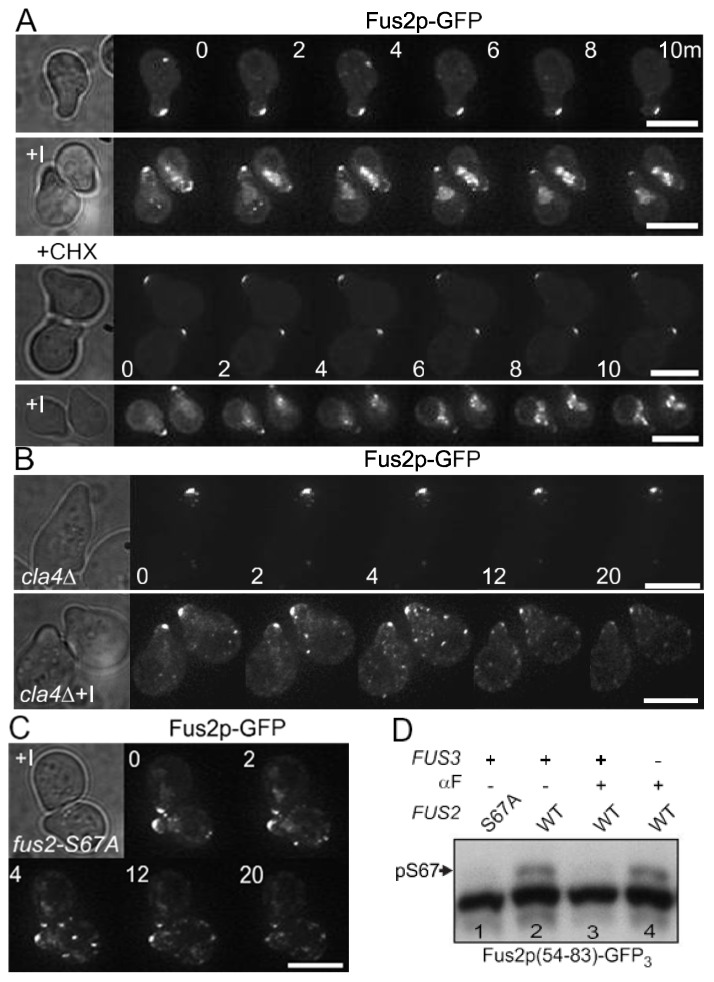
Inhibition of Fus2-S67 phosphorylation by Fus3p in mating cells. (**A**–**C**) The localization of Fus2p-GFP was examined over time in *fus3-Q93G* (*fus3-as1*) as described in the Materials and Methods. Indicated strains were pre-grown in selective medium and induced with α-factor for 90 min. Images were collected immediately after addition of the inhibitor (1-NA-PP1) at t = 0 and at 1 min intervals. Bar, 5 µm. (**A**) Wild-type (MY15342) containing the *fus3-as1* allele was observed with (+I) and without inhibitor. For the bottom two panels, cycloheximide (CHX) was added to a final concentration of 35 µg/mL, 15 min before inhibitor treatment. (**B**) A *cla4*Δ *fus3-as1* mutant (MY15305) was observed. (**C**) A *fus2-S67A fus3-as1* mutant (MY15123) was observed. (**D**) Fus2p^54−83^- or Fus2p^54−83^-S67A-GFP_3_ was expressed under control of the *GAL1* promoter in *FUS3* (MY12553 and MY12300) or *fus3*Δ *cln3*Δ (MY12282) for 90 min during mitotic growth or after α-factor arrest for 2 h. Samples were run on 50 µM Phos-tag gels and detected by anti-GFP. Arrow indicates the phosphorylated species (pS67).

Nuclear accumulated Fus2p is exported to the cytoplasm where it localizes to the shmoo-tip and cytoplasmic puncta after cytokinesis [25]. Using an inhibitor-sensitive mutant, *fus3-as1* (*fus3-Q93G*) [32,33], previous work showed that treatment with a selective kinase inhibitor, 1-NA-PP1, caused rapid changes in Fus2p-GFP localization [26]. Concomitant with decreased localization at the shmoo-tip, Fus2p-GFP becomes strongly localized in the nucleus. In principle, the change in localization could result either from pre-existing Fus2p moving from the shmoo-tip to the nucleus, or, alternatively, cytoplasmic Fus2p might be degraded and nuclear Fus2p-GFP might consist of newly synthesized protein that is sequestered in the nucleus. To distinguish between these possibilities, relocalization was examined in the presence of cycloheximide (CHX) to block protein synthesis. As reported previously (Figure 1A, top), Fus2p-GFP relocalized rapidly from the shmoo-tip to the nucleus in response to inhibition of Fus3p, but remained at the shmoo-tip in the control. Cycloheximide had no effect on the cellular distribution of Fus2p-GFP (Figure 1A, bottom). After addition of the inhibitor, Fus2p-GFP rapidly relocalized to the nucleus similar to the kinetics without cycloheximide. Therefore, the Fus2p that relocalized to the nucleus was pre-existing, implying that Fus3p kinase activity is required to actively maintain Fus2p in the cytoplasm, preventing re-import into the nucleus during mating.

We next asked whether the import of Fus2p is dependent on Cla4p and Fus2p-S67 phosphorylation, as it is when it is newly synthesized during mitosis. The *cla4*Δ cells are somewhat more elongated than wild-type cells due to disrupted septin assembly. Nevertheless, the *cla4*Δ strain formed normal pear-shaped pheromone-arrested cells in which Fus2p-GFP was localized at the shmoo-tip (Figure 1B). After addition of the inhibitor, Fus2p-GFP disappeared from the shmoo-tip, similar to wild-type. However, relocalization to the nucleus was not observed in the *cla4*Δ cells; instead, Fus2p-GFP was found in puncta randomly distributed in the cytoplasm. Similar diffuse cytoplasmic localization was observed in *CLA4+* cells in which Fus2p-S67 was mutated to alanine to prevent phosphorylation by Cla4p (Figure 1C). These results suggest that Fus3p kinase activity might prevent Fus2p-S67 from being phosphorylated by Cla4p, which would be sufficient to block the relocalization of Fus2p to the nucleus during mating. Furthermore, because Fus2p-GFP disappears from the shmoo-tip in either case, we conclude that Fus2p’s cortical localization is independent of Cla4p, and solely reflects Fus3p regulation. 

To examine directly whether Fus3p can block S67 phosphorylation, Fus2p^54−83^-GFP_3_ was ectopically expressed under control of the *GAL* promoter (Figure 1D). Fus2p^54−83^ contains only a single phosphorylation site, S67, the target of Cla4p. Triple-GFP (GFP_3_, 80 kDa) was used to prevent passive diffusion into the nucleus, as occurs for proteins <40 kDa. As previously shown [24], Phos-tag polyacrylamide enhanced the mobility shift of the phosphorylated protein in mitotic cells (Figure 1D, lane 2), which was absent in an S67A mutant (Figure 1D, lane 1). The S67 phosphorylation band was strongly reduced in pheromone-arrested wild-type cells (Figure 1D, lane 3), but present in pheromone-treated *fus3*Δ mutant cells (Figure 1D, lane 4). Note that the *fus3*Δ mutant also contained *cln3*Δ, which allows for cell cycle arrest in pheromone [34]. Thus, the phosphorylation of Fus2p-S67 by Cla4p is down-regulated by activated Fus3p, which helps maintain Fus2p in the cytoplasm as cells enter the mating pathway.

### 3.2. Cla4p-S186 and -S425 Are Phosphorylated Specifically by Fus3p 

**Figure 2:**
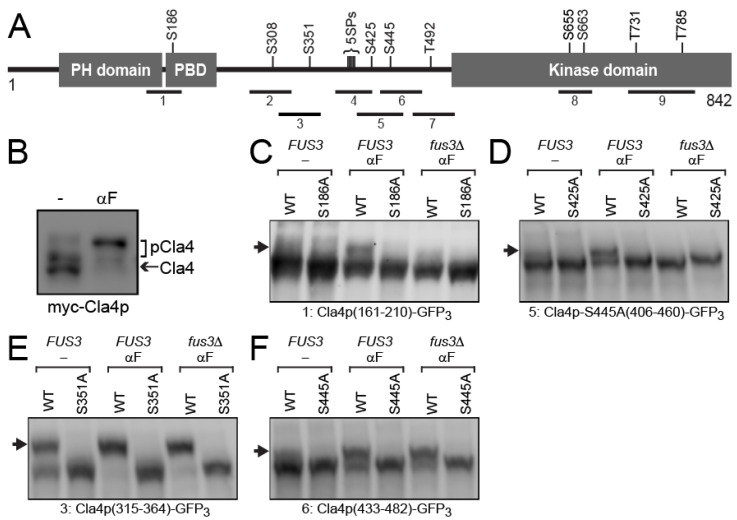
Screening for mating-specific and Fus3p-dependent phosphorylation sites in Cla4p. (**A**) Schematic representation of Cla4p. The PH (Pleckstrin Homology, 64–175 aa), PBD (p21-Rho-binding domain, 184–242 aa), and Kinase domain (517–842 aa) are indicated. The Cla4p fragments examined in this study and the positions of the 15 proline-directed serines (SP) or threonines (TP) are indicated. (**B**) Hyper-phosphorylation of Cla4p during mating. The myc-Cla4p was expressed in a *cla4*Δ strain (MY13001) for 2 h, plus or minus pheromone, electrophoresed on a 50 µM Phos-tag gel, and detected with anti-myc. The bracket marked “pCla4” indicates phosphorylated species. (**C**–**F**) The indicated truncations of Cla4p, fused to GFP_3_ were expressed in *FUS3* (MY11198) or *fus3*Δ *cln3*Δ (MY10273) under control of the *GAL1* promoter for 90 min during mitosis or after α-factor arrest for 2 h. Samples were run on 50 µM Phos-tag gels and detected by anti-GFP. Arrowheads indicate phosphorylated species. (**C**–**F**) Indicated truncations on plasmids were expressed. (**C**) pMR6414 and pMR 6415, (**D**) pMR6426 and pMR6427, (**E**) pMR6289 and pMR6290, (**F**) pMR6291 and pMR6292. (**C**–**F**) Arrows indicate phosphorylated species.

The PAK kinase Cla4p carries an N-terminal PH (Pleckstrin Homology), PBD (p21-Rho-binding domain) and C-terminal protein kinase domain (Figure 2A). Hyper-phosphorylation of Cla4p during mitosis is required for the phosphorylation and activation of one of its substrates, the Gin4p kinase [23]. To gain insight into the regulation of Cla4p’s phosphorylation of Fus2p, we examined whether the phosphorylation of Cla4p is also regulated by pheromone. To ensure similar levels of expression, Cla4p was expressed under control of the *GAL1* promoter, in the presence or absence of pheromone (Figure 2B). In mitotic cells, electrophoresis in Phos-tag polyacrylamide showed Cla4p to comprise at least three species, due to different levels of phosphorylation. The faint upper band became much more abundant after pheromone treatment, while the major lower bands were largely eliminated, indicating that Cla4p is much more strongly phosphorylated during mating than during mitosis. This led us to hypothesize that Fus3p might phosphorylate Cla4p to down-regulate Cla4p kinase activity and Fus2p-S67 phosphorylation.

Cla4p carries fifteen serine or threonine residues adjacent to prolines (SP or TP), which are potential substrates for proline-directed protein kinases (e.g., CDK/cyclin and MAP kinases, such as Fus3p) (Figure 2A). To identify mating- and/or Fus3p-specific phosphorylation site(s), we examined different fragments of Cla4p, each of which has one or two SP/TP sites (see Table S1 in the Supplementary Materials) and examined their phosphorylation status using Phos-tag gel electrophoresis. One additional fragment (#4) carried a cluster of five SP sites. Each fragment was N-terminally fused to GFP_3_ to facilitate analysis. To confirm that any mobility shift was due to phosphorylation of the indicated residue, each candidate serine or threonine was mutated to alanine. The hybrid proteins were transformed into *FUS3* or *fus3*Δ cells and expressed under control of the *GAL1* promoter in the presence or absence of pheromone. The *fus3*Δ strains also contained *cln3*Δ to facilitate G1 arrest in the presence of pheromone, and the *cdc28-as1* allele, an analogue-sensitive mutant of Cdc28p, to allow investigation of phosphorylation events by the yeast CDK during mitosis [32].

Four SP sites were observed to be phosphorylated (or hyper-phosphorylated) in response to pheromone. Fragment 1, containing S186, was detected as a single broad band in asynchronous mitotic cells, but gave rise to a distinct slower migrating species after pheromone treatment (Figure 2C). The slower migrating species was abolished either by *FUS3* deletion or by mutation of S186 to alanine (Figure 2C). Similarly, fragment 5, containing S425, was phosphorylated in a Fus3p and pheromone-dependent manner (Figure 2D). Note that, because this fragment would contain two SP sites (S425 and S445), S445 was also mutated to alanine to assess phosphorylation specifically at S425 (Figure 2D). 

Fragment 3, containing S351, exhibited both a slower and a faster migrating band in the absence of pheromone; the faster species was completely gone after pheromone treatment (Figure 2E). This suggests that Cla4p-S351 is phosphorylated partially in vegetative growing cells and fully in mating cells. Although the S351A mutation abolished the phosphorylation, deletion of *FUS3* had no significant effect (Figure 2E). Finally, fragment 6, containing only S445, also showed pheromone-specific phosphorylation, which was not affected by *fus3*Δ, suggesting that it is not likely to be relevant to Cla4p’s down-regulation by Fus3p (Figure 2F). All other truncations showed no evidence of slower migrating bands in the presence or absence of pheromone (Figure S1). In sum, we conclude that during mating, exposure to pheromone causes Cla4p to be hyper-phosphorylated at multiple sites. Of these, two sites, S186 and S424, are specific for Fus3p.

### 3.3. MAP-Kinase Slt2p Phosphorylates Cla4p-S351

To identify the specific kinase(s) responsible for Cla4p-S351 and S445 phosphorylation, we examined proline-directed protein kinases other than Fus3p. At least 10 protein kinases in budding yeast have been predicted or shown to phosphorylate serine or threonine in a proline-directed manner. These include the cyclin-dependent protein kinases (CDKs) Cdc28p and Pho85p, the CDK-like Yak1p, and six MAP-kinases (Fus3p, Kss1p, Slt2p, Smk1p, Hog1p, and Kdx1p). Rim11p (Gsk3p) is also classed as proline-directed kinase [35], although this has not been observed [36]. Pho85p is a nutrient-sensitive CDK that associates with two different G1-cyclins, Pcl1p and Pcl2p; deletions in the cyclin genes down-regulate Pho85p kinase activity [37,38].

**Figure 3:**
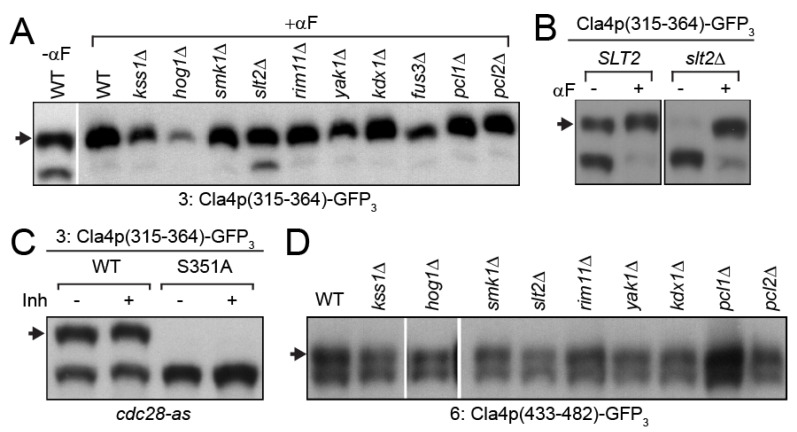
Screening for kinases responsible for Cla4p-S351 and S445. (**A**–**D**) Samples were run on 50 µM Phos-tag gels and detected by anti-GFP immunoblotting. Arrows indicate phosphorylated species. (**A**) The Cla4p truncation (MR6289) was expressed in indicated mutant cells (MY10273, 11198, 13003, 13004, 13005, 13006, 13007, 13010, 13011, 13012, and 13013) under the *GAL1* promoter for 90 min after α-factor arrest for 2 h. (**B**) The indicated Cla4p truncation (MR6289 and 6290) was expressed in *SLT2* (MY11198) and *slt2*Δ (MY13011) under the *GAL1* promoter for 90 min during mitosis or after α-factor arrest for 2 h. (**C**) Actively growing *cdc28-as1* (MY11198) was treated with DMSO or the inhibitor, 1-NM-PP1, after which truncated proteins (MR6289 and 6290) were induced by the *GAL1* promoter for 90 min. (**D**) The Cla4p truncation (MR6291) was expressed in indicated mutant cells (MY11198, 13018, 13019, 13020, 13021, 13022, 13025, 13026, 13027 and 13028) under the *GAL1* promoter for 90 min after α-factor arrest for 2 h.

Fragment 3, containing S351, was observed to produce two species in mitosis, a slower and a faster migrating band; pheromone led to a strong reduction in the faster one (Figure 2E). Of the kinases tested, only deletion of *SLT2* had an effect on Cla4p-S351 phosphorylation; pheromone-treated *slt2*Δ cells showed increased levels of the faster migrating band, indicating reduced levels of phosphorylation of S351 (Figure 3A). Slt2p is the MAP-kinase that is activated by the cell-wall integrity pathway. During mitotic growth, deletion of *SLT2* completely abolished the slower migrating band (Figure 3B). These results suggest that Slt2p phosphorylates Cla4p-S351 during mitotic growth, but as cells enter the mating pathway, another protein kinase(s) hyper-phosphorylates S351. The mitotic CDK Cdc28p has been reported to be responsible for mitosis-specific hyper-phosphorylation of Cla4p, and S351 was the only SP/TP site that was observed to be phosphorylated during mitosis in this screen. To determine whether S351 phosphorylation is regulated by the mitotic CDK, we used the *cdc28-as1* allele that is highly sensitive to the inhibitor, 1-NM-PP1 [32]. Inhibitor treatment had no effect on the mitotic phosphorylation fragment-3, indicating that phosphorylation of Cla4p-S351 is CDK-independent (Figure 3C). 

Next, Cla4p-S445 phosphorylation was examined in the presence of pheromone. In all of the mutants, fragment 6 was phosphorylated to a similar extent as wild-type (Figure 3D). One possibility is that S445 is phosphorylated by more than one of the known proline-directed protein kinases. 

### 3.4. Phosphorylation of Cla4p-S186 Negatively Regulates Cla4p Kinase Activity In Vitro

**Figure 4:**
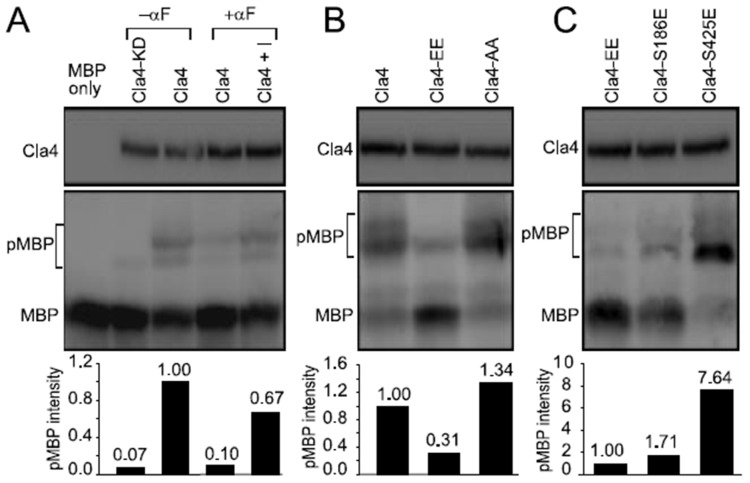
In vitro phosphorylation of MBP by Cla4p. In vitro kinase assays were performed as described in the Materials and Methods. The intensity of the slower-migrating MBP was normalized by the level of immunoprecipitated Cla4p and expressed relative to wild-type Cla4p (**A**,**B**) or Cla4p-EE (**C**). pMBP indicates phosphorylated MBP. (**A**) Cla4p or Cla4p-KD in the *fus3-as1* mutant was purified from mitotic cultures or cells treated with pheromone (MY15342 and 15343) for 2 h in the absence or presence of the inhibitor, 1-NA-PP1. (**B**) Wild-type, a phosphomimetic mutant Cla4p-EE (S186E, S425E), and the non-phosphorylatable mutant Cla4p-AA (S186A, S425A) were purified from mitotic extracts (MY15342, 15351, and 15647). (**C**) Cla4p single and double phosphomimetic mutants were purified from mitotic extracts (MY 15351, 15345, and 15349).

We next focused on the Fus3p and mating-dependent phosphorylation of S186 and S425 to assess their biological roles. To determine if S186 or S425 phosphorylation regulates Cla4p kinase activity, we performed in vitro kinase assays with affinity-purified Cla4p-HA and unphosphorylated myelin basic protein (MBP), which is an efficient PAK substrate [5]. As shown in Figure 4A, several slower-migrating species of MBP were produced after incubation with Cla4p immunoprecipitated from mitotic extracts (lane 3). The slower species were almost completely abolished using a kinase-dead version of Cla4p, Cla4-KD (lane 2), indicating that the mobility shifts are due to phosphorylation by Cla4p. Pheromone treatment reduced kinase activity over 10-fold, although equivalent amounts of Cla4p were immunoprecipitated (lane 4). When Fus3p kinase activity was inhibited by 1-NA-PP1, Cla4p kinase activity in the pheromone-treated cells remained high, at ~67% of the level in mitotic cells (lane 5). 

To determine if the pheromone-dependent down-regulation of Cla4p activity is due to Fus3p-dependent phosphorylation, S186 and S425 were mutated to glutamate (Cla4p-EE) or alanine (Cla4p-AA). The non-phosphorylatable Cla4p-AA immunoprecipitated from mitotic cells showed activity comparable to wild-type (Figure 4B). In contrast, the kinase activity of the phosphomimetic Cla4p-EE was decreased to ~30% of wild-type (Figure 4B). 

Examining the individual phosphomimetic mutants, the mitotic kinase activity of Cla4p-S186E was strongly reduced, only slightly higher than Cla4p-EE, whereas Cla4p-S425E retained essentially wild-type levels of activity (Figure 4C). Altogether, we conclude that Fus3p negatively regulates Cla4p kinase activity by inhibitory phosphorylation; S186 phosphorylation is the major site of regulation and S425 makes only a minor contribution.

### 3.5. Cla4p-S186 Phosphorylation Regulates Fus2p Phosphorylation and Nuclear Localization In Vivo

**Figure 5:**
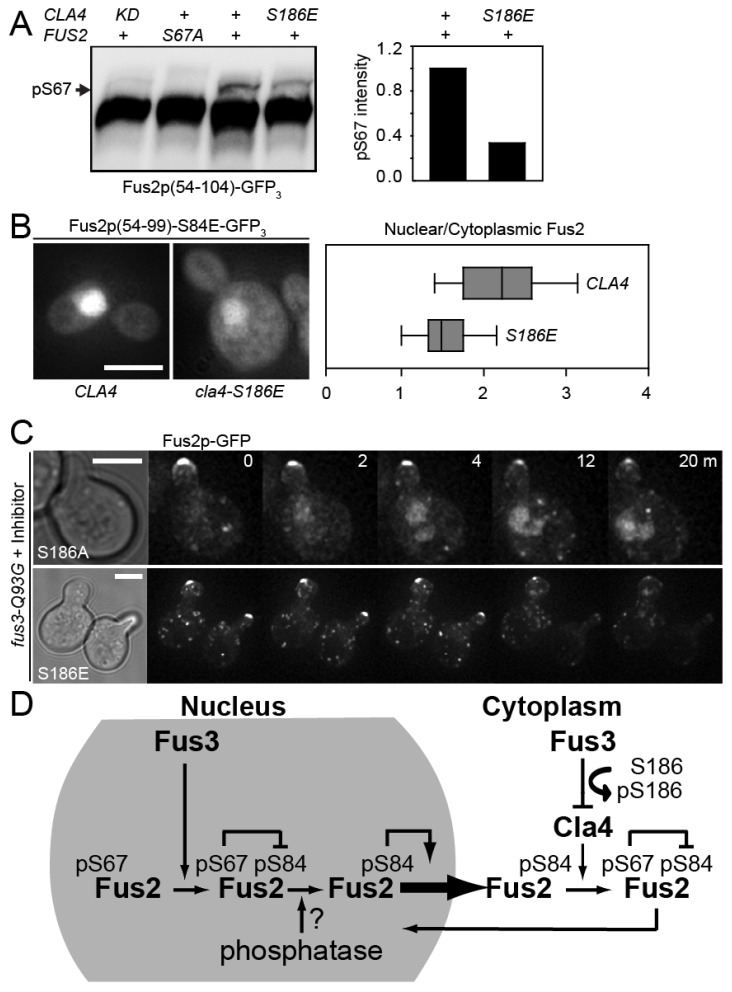
In vivo effects of Cla4-S186 phosphorylation. (**A**) Fus2p^54−104^-GFP_3_ (MR6009) and Fus2p^54−104^-S67A-GFP_3_ (MR6166) were expressed from the *GAL1* promoter for 90 min in strains MY15363, 15364, 15365, and 15366. Samples were separated on a 50 µM Phos-tag gel and detected by anti-GFP. The S67 phosphorylated species (arrow) was quantified relative to wild-type Cla4p (right panel). (**B**) Fus2p^54−99^-S84E-GFP_3_ (MR6160) was expressed from the *GAL1* promoter for 90 min in MY15355 and 15356. The intensity of nuclear fluorescence relative to an equal cytoplasmic area was measured in live cells (n = 52 for the *cla4-S186E* mutant and 181 for the *CLA4* wild-type), as described in the Materials and Methods. (**C**) Full length Fus2p-GFP was examined over time in *fus3-as1* mutant as in Figure 1A. MY15344 and MY15345 were induced with α-factor for 90 min; images were collected at 1 min intervals after inhibitor (1-NA-PP1) was added (t = 0). Bar, 5 µm. (**D**) A model for regulated Fus2p localization. Cla4p phosphorylation of Fus2p-S67 is required for nuclear localization; Cla4p kinase activity is down-regulated by activated Fus3p kinase. Fus3p also phosphorylates Fus2p-S84 to activate nuclear export. See the Discussion for detail.

To examine the in vivo consequences of Cla4p-S186 phosphorylation, we focused on the phosphorylation of one of its mitotic substrates, Fus2p-S67 (Figure 5). Hybrid proteins bearing fragments of the N-terminal domain that regulates nuclear localization (Fus2p^54−104^ or Fus2p^54−99^) were ectopically expressed in mitotic cells under control of the *GAL1* promoter in wild-type *CLA4* or mutant cells. S67 phosphorylation results in a slower migrating species in wild-type, but not in a *CLA4* kinase-dead mutant or when S67 was mutated to alanine (Figure 5A). In the *CLA4-S186E* mutant, the amount of phosphorylated Fus2p was reduced to ~37% of wild-type *CLA4* (Figure 5A), consistent with the in vitro assay showing that Cla4p-EE retained ~30% activity (Figure 4B). The in vivo effect of the reduction in S67 phosphorylation by Cla4p-S186E was revealed by examining the nuclear localization of Fus2p^54−99^-S84E (Figure 5B). Previous work demonstrated that the localization of this protein in mitotic cells is particularly sensitive to the level of S67 phosphorylation [24]. The phosphomimetic S84E mutation activates the Fus2p NES, which counters the dominant activation of the NLS by S67 phosphorylation [24]. The ratio of nuclear to cytoplasmic fluorescence intensity was significantly reduced in the *cla4-S186E* mutant, 1.52 ± 0.34, compared to the wild-type *CLA4*, 2.23 ± 0.65 (n = 52 and 181, respectively, *p* = 2 × 10^−12^, Mann–Whitney test).

We next examined the relocalization of full-length Fus2p-GFP to the nucleus following inhibition of Fus3p activity. Inhibition of Fus3p caused rapid loss of Fus2p-GFP from the shmoo-tip in both *cla4-S186A* and *cla4-S186E* mutants (Figure 5C), similar to the kinetics observed for wild-type *CLA4* (Figure 1A). In the *cla4-S186A* mutant, Fus2p-GFP accumulated in the nucleus, similar to wild-type *CLA4*. However, in the *cla4-S186E* mutant, Fus2p-GFP failed to localize in the nucleus, showing only diffuse and punctate cytoplasmic localization, as was observed for *cla4*Δ and *fus2-S67A* cells (Compare Figure 5C and Figure 1B,C). Taken together, we conclude that Cla4p kinase activity is down-regulated by inhibitory phosphorylation by Fus3p at S186. Inhibition of Cla4p is required to help maintain Fus2p in the cytoplasm to regulate cell wall removal during mating (Figure 5D). 

### 3.6. Biological Function of the Inhibitory Phosphorylation of Cla4p by Fus3p Is Not Limited to Fus2p Localization 

**Figure 6:**
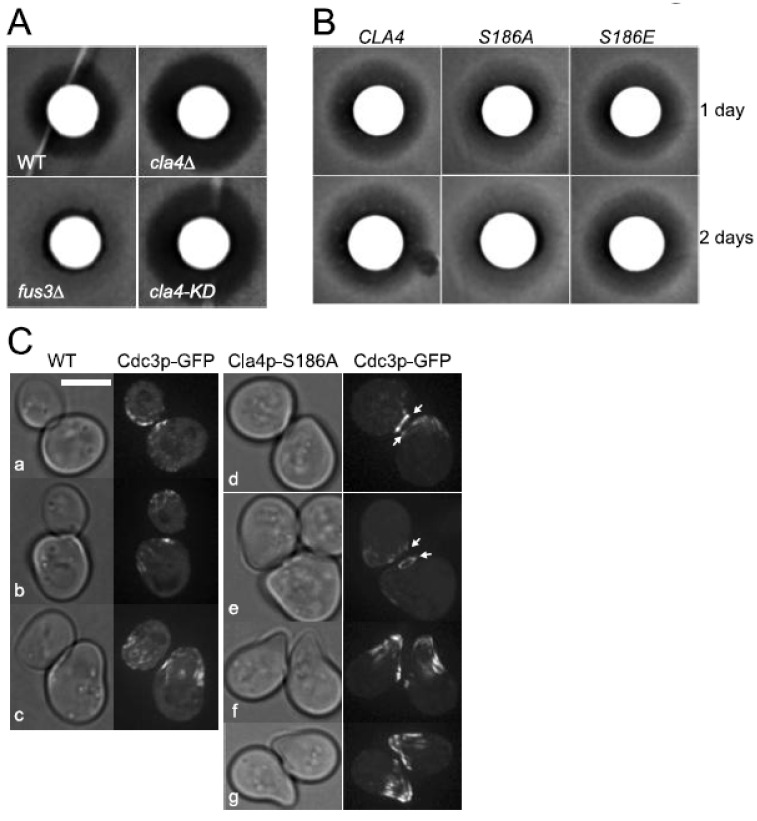
Pheromone sensitivity, recovery, and mating-specific septin structure in *cla4* mutants. (**A**,**B**) Cla4p regulates sensitivity to pheromone; Fus3p phosphorylation of Cla4p delays recovery from pheromone; 10 µg of α-factor was spotted on filter disks on a thin layer of cells and incubated at 30 °C for 1 (**A**,**B**) to 2 days (**B**). (**A**) Halo assays of MY15308, MY15311, MY15310, and MY15309. (**B**) Halo assays of MY15342, MY15344 and MY15345. (**C**) Cdc3p-GFP (MR5142) were observed 1 h after α-factor was added in wild-type and *CLA4-S186A* cells (MY15953 and 15951) were observed 1 h after α-factor was added. Arrows indicate a mitotic septin ring. Bar, 5 µm.

A potential function for Cla4p as a negative regulator of the response to pheromone was suggested by several pheromone-resistant variants of Cdc42p that showed reduced binding to Cla4p [39,40]. Moreover, overexpression of Cla4p was sufficient to confer resistance to pheromone-induced cell cycle arrest [40]. Thus, we examined whether loss of the inhibitory phosphorylation by Fus3p can modulate the response to pheromone of the *cla4* phosphorylation site mutants. A halo assay, in which pheromone is applied to a filter disk in the middle of a lawn of cells, is a sensitive measure of the cellular response to pheromone (Figure 6A,B). The *cla4*Δ was significantly more sensitive to pheromone than wild-type (Figure 6A). The *cla4-KD* mutant exhibited a zone of inhibition similar to the *cla4*Δ, implying that Cla4p kinase activity antagonizes the pheromone response (Figure 6A). Both the *S186E* and *S186A* mutants displayed wild-type-sized zones of growth inhibition. Growth remained inhibited over an extended period of time for both the wild-type and *S186E* mutant. However, for the non-phosphorylatable *cla4-S186A* mutant, the inhibition zone became partially filled in with cell growth, leading to a turbid halo (Figure 6B). This indicates that the *cla4-S186A* mutant recovered more rapidly from pheromone inhibition, suggesting that down-regulation of Cla4p by Fus3p is required to maintain pheromone-dependent cell cycle arrest.

Cla4p kinase activity is required for organizing an hourglass-shaped septin collar at the mother-bud neck that splits two rings during cytokinesis [17]. The septin rings disassemble prior to the next round of cell cycle, and then reappear at the presumptive bud site [41,42,43]. In response to pheromone, cells relocalize the septins into a diffuse band of filaments at the neck of shmoo, aligned with the long axis of the cell [44] (Figure 6C, b and c). The wide band of septin filaments is similar in appearance to mitotic cells that lack Cla4p. Thus, we examined the septin localization in the *cla4-S186A* mutant. No differences were observed for the mitotic septin structure comparing wild-type and *cla4-S186A*. Instead, septin ring disassembly after cytokinesis was delayed in the pheromone-treated *cla4-S186A* mutant (Figure 6C, d and e). In wild-type cells treated with pheromone, the septin ring rapidly disappeared such that cells with a mating projection only contained the band of septin filaments at the shmoo neck (Figure 6C, a and b). In contrast, most of the *cla4-S186A* mutant cells contained remnant septin rings at the previous cleavage site, as well as the band of septin filaments at the shmoo neck (Figure 6C, d and e). Over time, the septin ring eventually disappeared in the *cla4-S186A* mutant cells, although they typically retained septin at the tip of shmoo projection. In addition, the mating projection appeared to be more highly polarized than the wild-type cell (Figure 6C, f and g). This result suggests that the inhibition of Cla4p kinase activity by Fus3p affected the timing and formation of the mating-specific septin relocalization. Taken together, we conclude that the balance between Cla4p and Fus3p protein kinase activities plays a general role in regulating the transition between mating and mitosis.

## 4. Discussion

Cellular proliferation and differentiation are mutually exclusive programs, which are carefully coordinated during development. In the budding yeast *Saccharomyces cerevisiae*, a pheromone-responsive mitogen-activated protein kinase (MAPK) cascade regulates the differentiation to form specialized cells that are capable of cell and nuclear fusion [45]. Because of the potential for conflicting cellular programs during the initial response to pheromone, vegetatively growing cells that are still in mitosis regulate mating to ensure the orderly completion of the cell cycle. One known mechanism is by regulating subcellular localization of a key mating regulator, Fus2p, that acts together with the GTPase Cdc42p to activate cell fusion at the interface between mating cells [24,26,28,29]. Upon initial exposure to pheromone, mitotic cells induce Fus2p, but sequester it in the nucleus. Nuclear localization is due to Cla4p phosphorylation of Fus2p-S67 activating an NLS sequence that is epistatic to signals for nuclear exit [24]. As cells exit the cell cycle, CDK and Cla4p kinase activity are reduced, and Fus3p phosphorylation of Fus2p-S84 and S100 activate an NES and promotes nuclear export [24,28]. The export of Fus2p from the nucleus suggests that an as-yet unidentified phosphatase reverses Fus2p-S67 phosphorylation to allow Fus2p exit after cytokinesis [28]. After Fus2p is cytoplasmic, Fus3p kinase activity was found to be required for maintaining localization at the shmoo-tip (Figure 5D). Most significantly, we find that, during mating, Fus3p inhibits Cla4p kinase activity via the phosphorylation of S186, which blocks the phosphorylation of Fus2p-S67 and relocalization to the nucleus. This report is the first to demonstrate a regulatory mechanism for this important PAK kinase, Cla4p. Fus3p and Cla4p function as counteracting kinases with respect to Fus2p.

The binding of the GTP-bound form of Cdc42p has been proposed to open the inactive conformation of Cla4p [5]. It is interesting that S186 is within the PBD domain in Cla4p (Figure 2A). Thus, one possible mechanism is that phosphorylation could modulate the binding activity of the PBD domain to Cdc42p. However, the phosphomimetic mutation Cla4p-S186E did not disrupt the interaction with Cdc42p using a yeast two-hybrid assay, either in full-length Cla4p or the isolated PBD (see Figure S2). It is possible that increased levels of the proteins in the two-hybrid assay effectively masked any decreased affinity. Alternatively, phosphorylation of S186 may only affect the allosteric relief of auto-inhibition that occurs upon Cdc42p-binding. Interestingly, full-length Cla4p showed stronger activation of the reporter gene than the PBD, suggesting that full-length Cla4p is required for efficient Cdc42p binding and/or Cla4p might carry additional Cdc42p-binding motifs. Further studies are needed to elucidate how S186 phosphorylation inhibits the kinase activity of Cla4p, which may shed light on additional mechanisms of PAK kinase regulation.

Cla4p exhibits mitotic specific hyper-phosphorylation that is associated with GTP-bound Cdc42p and the Clb2p–Cdc28 kinase complex [23]. However, our screening showed that only S351 was strongly phosphorylated during mitosis and this phosphorylation was independent of mitotic CDK (Figure 2 and Figure 3). None of the SP or TP sites showed high levels of phosphorylation in asynchronous mitotic cultures (Figure 2 and Figure S1). Thus, it is still unknown whether mitotic CDK directly phosphorylates Cla4p. It is possible that full-length Cla4p may be required for mitotic phosphorylation by the Clb2p–Cdc28p kinase. Alternatively, mitotic CDK might contribute to Cla4p phosphorylation indirectly and a different kinase may be responsible. For example, deletion of the Elm1p kinase has been reported to significantly reduce mitotic hyper-phosphorylation of Cla4p [46]. In addition, we showed the MAP-kinase Slt2p phosphorylates Cla4p. The biological function of S351 phosphorylation by Slt2p is unclear, but several previous genetic screens demonstrated synthetic–lethal interactions between *CLA4* and *SLT2* mutations [47,48,49]. Interestingly, both proteins are implicated in morphogenesis; the MAP kinase Slt2p is important for cell wall integrity during polarized growth [50,51], whereas Cla4p has been proposed to be part of a negative feedback loop that down-regulates Cdc24p to turn off polarized growth [13]. These observations tempt us to propose that the polarized cell growth might be integrated into the cell-wall integrity pathway via Cla4p and Slt2p. Previous large scale phosphoproteomic studies identified five phosphorylation sites, S186, S351, S425, S445, and T492 in mitotic cells [52,53,54]. Only three sites, S351, S425, and S445, were detected in all three studies; in one study, the phosphorylation was only observed in cells synchronized by mitotic arrest [53]. Therefore, it is likely that the phosphorylation of these sites in mitosis is either sub-stoichiometric or transient during the cell cycle. 

Previously, in vitro kinase assays with immunoprecipitated Cla4p showed that the kinase activity can be detected throughout cell cycle. As cells proceeded into mitosis, the kinase activity increased sevenfold, but dropped following cytokinesis and entry into the G1 phase [5]. Thus, it is likely that the differential kinase activity of Cla4p may correlate with various mitotic events from bud emergence to cytokinesis. Alternatively, Cla4p may have functions that are independent of its kinase activity. For example, overexpression of a truncated Cla4p deleted for the kinase domain can suppresses a *cdc24* allele that is defective in cell polarity [55]. In this study, we showed that pheromone treatment leads to a dramatic reduction in Cla4p kinase activity, comparable to a kinase-dead mutant. On the other hand, the kinase activity of Cla4p-EE, the Fus3p- and mating-dependent phosphomimetic mutant was only decreased about threefold in mitotic cells (Figure 4B). Consistent with this observation, there was a similar decrease in both Fus2p phosphorylation and nuclear localization in mitotic cells that express Cla4p-S186E (Figure 5A,B). These results suggest that the residual Cla4p kinase activity after Fus3p phosphorylation would be responsible for keeping Fus2p in the nucleus, until the completion of mitosis. As cells enter the mating pathway, Cla4p kinase activity might be suppressed on multiple levels, through a reduction in the stimulatory CDK activity and through increased inhibitory Fus3p activity.

In this paper, we demonstrated a novel mechanism by which the pheromone-activated MAP kinase Fus3p negatively regulates the PAK kinase Cla4p, using Fus2p as a readout of their activities. However, it is likely that Fus3p down-regulation of Cla4p is not limited to Fus2p. A Fus3p phosphorylation-defective *cla4* mutant showed more rapid recovery from mating arrest, suggesting that reactivation of Cla4p is part of the mechanism of recovery from pheromone arrest (Figure 6B). In addition, lack of Fus3p-dependent phosphorylation of Cla4p during the transitions to mating caused a delay in the disassembly of mitotic septin ring and the organization of mating-specific septin structure (Figure 6C). Interestingly, the *cla4-S186A* mutation also appeared to enhance the polarized growth of the mating projections. Loss of the inhibitory phosphorylation may disrupt the down-regulation of septin as a diffusion barrier for mating signaling and polarization components. Thus, the balance of mutually antagonistic Cla4p and Fus3p has a larger biological significance during the transitions between mitosis and mating. It is likely that similar processes occur during other differentiation events.

## Data Availability

All data presented in this study are available within the article or Supplementary Materials. All strains and plasmids are available upon request.

## Supplementary Materials

The following supporting information can be downloaded at: https://www.mdpi.com/article/10.3390/biom12040598/s1, Figure S1: Screening for mating-specific and Fus3p-dependent phosphorylation sites of Cla4p. Images showing screens for additional mating and Fus3p-dependent phosphorylation sites in Cla4 fragments. Figure S2: Interaction between Cla4p mutants and Cdc42p by yeast two-hybrid. The effect of Cla4-S186 phosphorylation on the interaction with Cdc42p was analyzed by yeast two-hybrid. Table S1: Yeast strains used in this study. Table S2: Plasmids used in this study.
