# Supplementary material for: Cla4p Kinase Activity Is Down-Regulated by Fus3p during Yeast Mating
Source: Biomolecules. 2022 Apr 18;12(4):598. doi: 10.3390/biom12040598 (PMC9028331; doi:10.3390/biom12040598)
Supplement: Supplementary file 1 [file biomolecules-12-00598-s001.zip › biomolecules-1679280-supplementary.pdf]

SUPPLEMENTAL MATERIALS

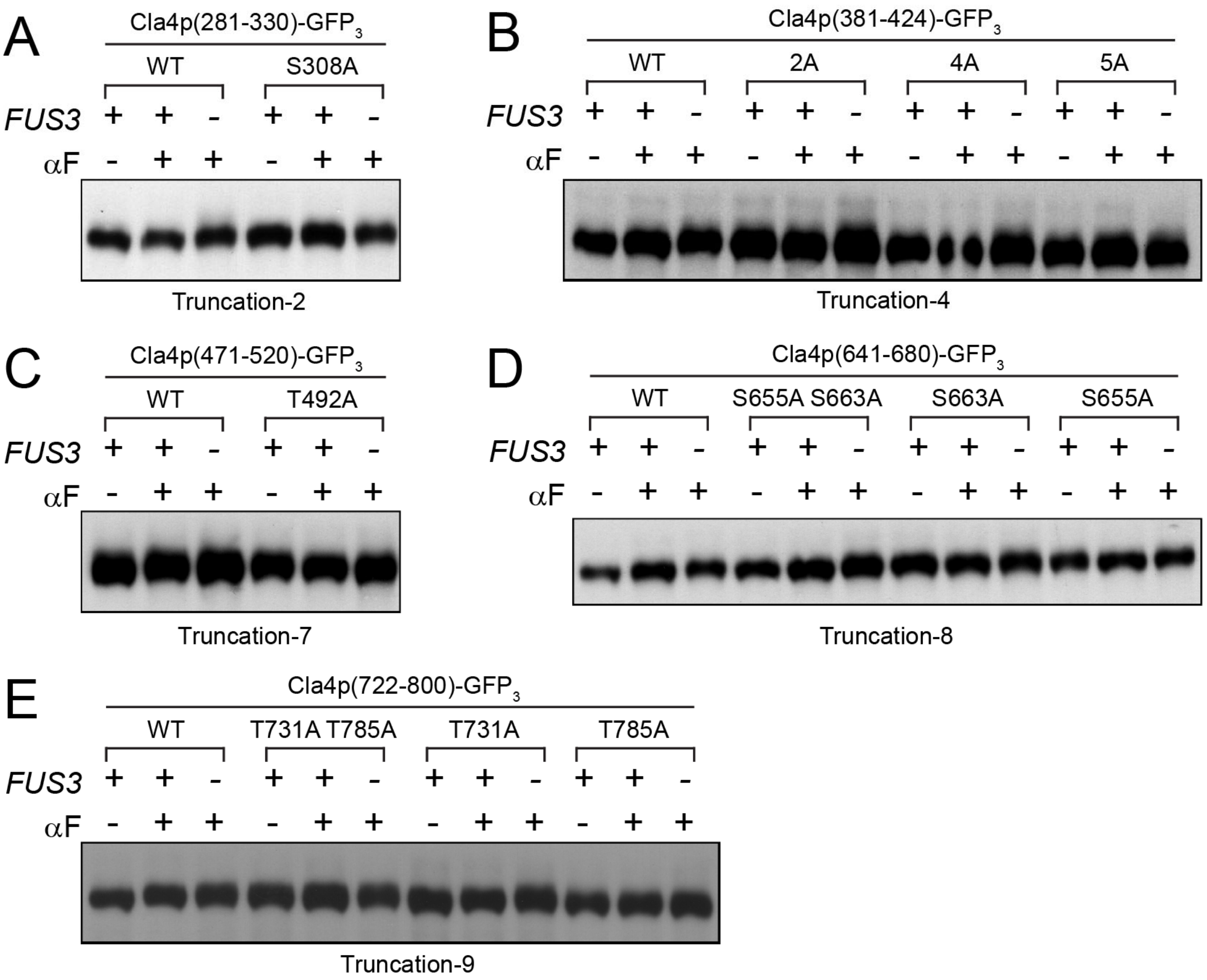

Figure S1. Screening for mating specific and Fus3p-dependent phosphorylation sites of Cla4p. Indicated fragments of Cla4p were expressed in *FUS3* (MY11198) or *fus3*Δ *cln3*Δ (MY10273) cells under the *GAL1* promoter for 90 min during mitosis or after α-factor arrest for 2 h as described in Fig. 2, C to F. Samples were run on 50 μM Phos-tag gels and detected by anti-GFP immunoblotting. (A) Indicated Cla4p fragments (MR6412 and 6413) were expressed. (B) Indicated Cla4p fragments (MR6428, 6429, 6430 and 6431) were expressed. (C) Indicated Clar4p fragments (MR6424 and 6425) were expressed. (D) Indicated Cla4p fragments (MR6416, 6417, 6418 and 6419) were expressed. (E) Indicated Cla4p fragments (MR6420, 6421, 6422 and 6423) were expressed.

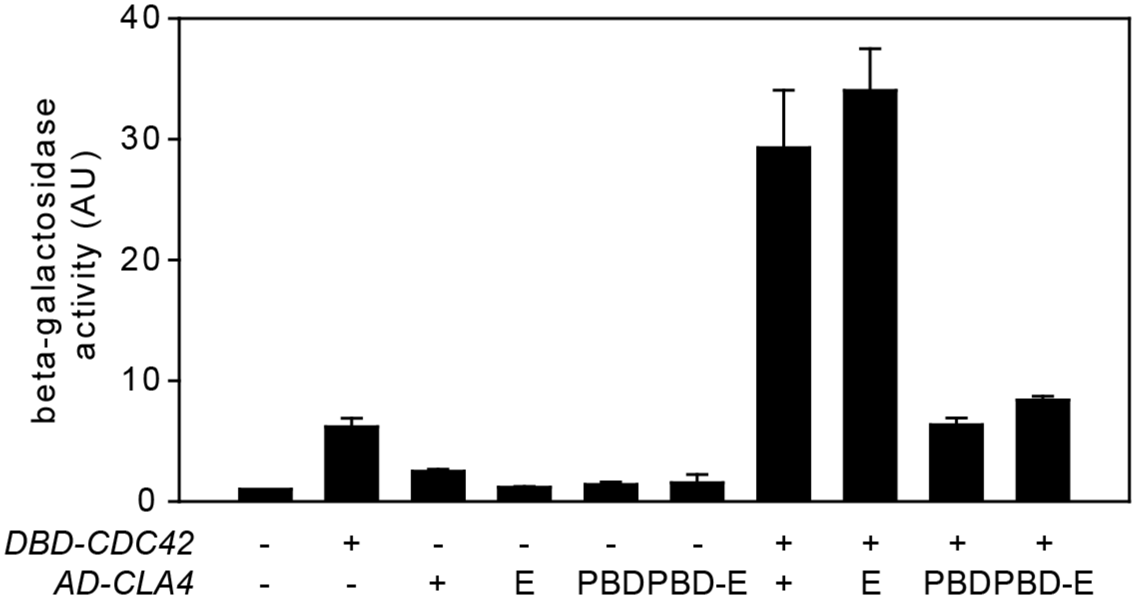

Figure S2. Interaction between Cla4p mutants and Cdc42p by yeast two-hybrid. The effect of Cla4-S186 phosphorylation on the interaction with Cdc42p was analyzed by yeast two-hybrid. The plasmids used in yeast two-hybrid are listed in Table S2. Cdc42p was fused to the DNA-binding domain (DBD) in pEG202. All Cdc42 hybrid proteins carry the C188S mutation to prevent localization to the plasma membrane. Cla4p was fused to the activation domain (AD) in pJG4-5. Full-length wild-type Cla4p, Cla4p-S186E, Cla4p-PBD domain (184-242), and Cla4p-PBD-S186E were fused to the activation domain (AD) in pJG4-5. The reporter strain EGY48-p1840 (MY15371) was co-transformed with pEG202 and pJG4-5 constructs. Transformants grown to mid-log in 2% raffinose were transferred to media with 2% galactose for 4h and then β-galactosidase activity was measured. All assays were performed in triplicate on three independent transformants for each combination of plasmids and plotted, mean ± SD. E, full-length Cla4p-S186E. PBD, Cla4p-PBD domain. PBD-E, Cla4p-PBD-S186E.

Supplemental Table S1. Yeast strains used in this study.

Strain	Genotype	Source*
BY4741 derivatives		
BY4741	*MAT***a** *ura3-Δ0 his3-Δ200 leu2-Δ0 met15-Δ0*	
MY10273	*fus2Δ::HIS3 fus3Δ::natMX cln3Δ::LEU2 CDC3-mCherry::kanMX*	
MY11198	*cdc28-as1*	
MY12282	*fus2Δ::HIS3 fus3Δ::natMX cln3Δ::LEU2 CDC3-mCherry::kanMX*<br>*MR6152* [P_{GAL1}-*fus2(54-83)*-GFP_3]	
MY12292	*fus2Δ::HIS3 fus3Δ::natMX cln3Δ::LEU2 CDC3-mCherry::kanMX*<br>*MR6289* [P_{GAL1}-*cla4(315-364)*-GFP_3]	
MY12300	*fus2Δ::HIS3 CDC3-mCherry::kanMX MR6152* [P_{GAL1}-*fus2(54-83)*-GFP_3]	
MY12553	*fus2Δ::HIS3 CDC3-mCherry::kanMX MR6153* [P_{GAL1}-*fus2(54-83)-S67A*-GFP_3]	
MY13001	*cla4Δ::natMX MR6194* [P_{GAL1}-*myc-CLA4*]	
MY13003	*rim11Δ::kanMX MR6289* [P_{GAL1}-*cla4(315-364)*-GFP_3]	
MY13004	*kss1Δ::kanMX MR6289* [P_{GAL1}-*cla4(315-364)*-GFP_3]	
MY13005	*smk1Δ::kanMX MR6289* [P_{GAL1}-*cla4(315-364)*-GFP_3]	
MY13006	*pcl1Δ ::kanMX MR6289* [P_{GAL1}-*cla4(315-364)*-GFP_3]	
MY13007	*pcl2Δ::kanMX MR6289* [P_{GAL1}-*cla4(315-364)*-GFP_3]	
MY13010	*hog1Δ::kanMX MR6289* [P_{GAL1}-*cla4(315-364)*-GFP_3]	
MY13011	*slt2Δ::kanMX MR6289* [P_{GAL1}-*cla4(315-364)*-GFP_3]	
MY13012	*yak1Δ::kanMX MR6289* [P_{GAL1}-*cla4(315-364)*-GFP_3]	
MY13013	*ykl161cΔ::kanMX MR6289* [P_{GAL1}-*cla4(315-364)*-GFP_3]	
MY13018	*rim11Δ::kanMX MR6291* [P_{GAL1}-*cla4(433-482)*- GFP_3]	
MY13019	*kss1Δ::kanMX MR6291* [P_{GAL1}-*cla4(433-482)*- GFP_3]	
MY13020	*smk1Δ::kanMX MR6291* [P_{GAL1}-*cla4(433-482)*- GFP_3]	
MY13021	*pcl1Δ::kanMX MR6291* [P_{GAL1}-*cla4(433-482)*- GFP_3]	
MY13022	*pcl2Δ::kanMX MR6291* [P_{GAL1}-*cla4(433-482)*- GFP_3]	
MY13025	*hog1Δ::kanMX MR6291* [P_{GAL1}-*cla4(433-482)*- GFP_3]	
MY13026	*slt2Δ::kanMX MR6291* [P_{GAL1}-*cla4(433-482)*- GFP_3]	
MY13027	*yak1Δ::kanMX MR6291* [P_{GAL1}-*cla4(433-482)*- GFP_3]	
MY13028	*ykl161cΔ::kanMX MR6291* [P_{GAL1}-*cla4(433-482)*- GFP_3]	
MY15123	*fus2Δ::kanMX fus3Δ::hphMX rvs161Δ::natMX*<br>*MR6962* [*fus2-S67A-GFP*] *MR4938* [*FLAG-fus3-as1*] *MR6955*[*RVS161-mCherry*]	
MY15137	*fus2Δ::kanMX fus3Δ::hphMX cla4Δ::natMX*	
MY15305	*fus2Δ::kanMX fus3Δ::hphMX cla4Δ::natMX*<br>*MR6998* [*FUS2::*GFP_{104}] *MR4938* [*FLAG-fus3-as1*]	
MY15308	*fus2Δ::kanMX fus3Δ::hphMX cla4Δ::natMX*<br>*MR6998* [*FUS2::*GFP_{104}] *MR4937* [*FLAG-FUS3*] *MR6266*[*CLA4*-HA_3]	
MY15309	*fus2Δ::kanMX fus3Δ::hphMX cla4Δ::natMX*<br>*MR6998* [*FUS2::*GFP_{104}] *MR4937* [*FLAG-FUS3*] *MR6275*[*cla4-KD*-HA_3]	
MY15310	*fus2Δ::kanMX fus3Δ::hphMX cla4Δ::natMX*<br>*MR6998* [*FUS2::*GFP_{104}] *MR1868* [*pRS416*] *MR6266*[*CLA4*-HA_3]	

MY15311	*fus2Δ::kanMX fus3Δ::hphMX cla4Δ::natMX*<br>*MR6998* [*FUS2::GFP*$_{104}$] *MR4937* [*FLAG-FUS3*] *MR1867*[*pRS415*]	
MY15342	*fus2Δ::kanMX fus3Δ::hphMX cla4Δ::natMX*<br>*MR6998* [*FUS2::GFP*$_{104}$] *MR4938* [*FLAG-fus3-as1*] *MR6266* [*CLA4-HA*$_{3}$]	
MY15343	*fus2Δ::kanMX fus3Δ::hphMX cla4Δ::natMX*<br>*MR6998* [*FUS2::GFP*$_{104}$] *MR4938* [*FLAG-fus3-as1*] *MR6275* [*cla4-KD-HA*$_{3}$]	
MY15344	*fus2Δ::kanMX fus3Δ::hphMX cla4Δ::natMX*<br>*MR6998* [*FUS2::GFP*$_{104}$] *MR4938* [*FLAG-fus3-as1*] *MR6343*[*cla4-S186A-HA*$_{3}$]	
MY15345	*fus2Δ::kanMX fus3Δ::hphMX cla4Δ::natMX*<br>*MR6998* [*FUS2::GFP*$_{104}$] *MR4938* [*FLAG-fus3-as1*] *MR6991* [*cla4-S186E-HA*$_{3}$]	
MY15345	*fus2Δ::kanMX fus3Δ::hphMX cla4Δ::natMX*<br>*MR6998* [*FUS2::GFP*$_{104}$] *MR4938* [*FLAG-fus3-as1*] *MR6991*[*cla4-S186E-HA*$_{3}$]	
MY15349	*fus2Δ::kanMX fus3Δ::hphMX cla4Δ::natMX*<br>*MR6998* [*FUS2::GFP*$_{104}$] *MR4938* [*FLAG-fus3-as1*] *MR6992* [*cla4-S425E-HA*$_{3}$]	
MY15351	*fus2Δ::kanMX fus3Δ::hphMX cla4Δ::natMX*<br>*MR6998* [*FUS2::GFP*$_{104}$] *MR4938* [*FLAG-fus3-as1*] *MR6994* [*cla4-EE-HA*$_{3}$]	
MY15355	*cdc28-as1 fus2Δ::HIS3 cla4Δ::kanMX*<br>*MR6160* [P_{GAL1}*-fus2(54-99)-GFP*$_{3}$] *MR6266* [*CLA4-HA*$_{3}$]	
MY15356	*cdc28-as1 fus2Δ::HIS3 cla4Δ::kanMX*<br>*MR6160* [P_{GAL1}*-fus2(54-99)-GFP*$_{3}$] *MR6991* [*cla4-S186E- HA*$_{3}$]	
MY15363	*cdc28-as1 fus2Δ::HIS3 cla4Δ::kanMX*<br>*MR6009* [P_{GAL1}*-fus2(54-104)-GFP*$_{3}$] *MR6266* [*CLA4-HA*$_{3}$]	
MY15364	*cdc28-as1 fus2Δ::HIS3 cla4Δ::kanMX*<br>*MR6166* [P_{GAL1}*-fus2(54-104)-S67A-GFP*$_{3}$] *MR6266* [*CLA4- HA*$_{3}$]	
MY15365	*cdc28-as1 fus2Δ::HIS3 cla4Δ::kanMX*<br>*MR6009* [P_{GAL1}*-fus2(54-104)-GFP*$_{3}$] *MR6991* [*cla4-S186E- HA*$_{3}$]	
MY15366	*cdc28-as1 fus2Δ::HIS3 cla4Δ::kanMX*<br>*MR6009* [P_{GAL1}*-fus2(54-104)-GFP*$_{3}$] *MR6275* [*cla4-KD- HA*$_{3}$]	
MY15647	*fus2Δ::kanMX fus3Δ::hphMX cla4Δ::natMX*<br>*MR6998* [*FUS2::GFP*$_{104}$] *MR4938* [*FLAG-fus3-as1*] *MR7080* [*cla4-AA- HA*$_{3}$]	
MY15951	*cla4Δ::kanMX MR6343* [*cla4-S186A-HA*$_{3}$] *MR5142* [*CDC3-GFP*]	
MY15953	*cla4Δ::kanMX MR6266* [*CLA-HA*$_{3}$] *MR5142* [*CDC3-GFP*]	
Yeast two-hybrid reporter strain		
MY15371	EGY48-p1840 *MATα ura3 his3 trp1 leu2 lexAop-lacZ-URA3*	1

*All of cells without source were constructed in this study.

Supplemental Table S2: Plasmids used in this study

Plasmid	*Description*	Source*
pMR1867	*pRS415 CEN LEU2*	1
pMR1868	*pRS416 CEN URA3*	1
pMR4937	*CEN URA3 FLAG-FUS3*	2
pMR4938	*CEN URA3 FLAG-fus3-Q93G (fus3-as1)*	2
pMR5142	*CEN URA3 CDC3-GFP*	
pMR6009	*CEN URA3* P_{GAL}*-fus2(54-104)-* GFP_3	3
pMR6152	*CEN URA3* P_{GAL}*-fus2(54-83)-* GFP_3	
pMR6153	*CEN URA3* P_{GAL}*-fus2(54-83)-S67A-* GFP_3	
pMR6160	*CEN URA3* P_{GAL}*-fus2(54-99)-S84E-* GFP_3	
pMR6166	*CEN URA3* P_{GAL}*-fus2(54-104)-S67A-* GFP_3	
pMR6194	*CEN URA3* P_{GAL}*-myc-CLA4*	4
pMR6266	*CEN LEU2 CLA4-* HA_3	
pMR6275	*CEN LEU2 cla4-K594A (cla4-KD)-* HA_3	
pMR6343	*CEN LEU2 cla4-S186A-* HA_3	
pMR6955	*CEN HIS3 RVS161-mCherry*	
pMR6962	*CEN LEU2 fus2-S67A::* GFP_{104}	
pMR6991	*CEN LEU2 cla4-S186E-* HA_3	
pMR6992	*CEN LEU2 cla4-S425E-* HA_3	
pMR6994	*CEN LEU2 cla4-EE (S186E S425E)-* HA_3	
pMR6998	*CEN HIS3 FUS2::* GFP_{104}	
pMR7080	*CEN LEU2 cla4-AA (S186A S425A)-* HA_3	
Screening for pheromone and Fus3p-specific phosphorylation sites in Cla4p		# of Truncation
pMR6415	*CEN URA3* P_{GAL}*-cla4(161-210)-* GFP_3	Truncation-1
pMR6414	*CEN URA3* P_{GAL}*-cla4(161-210)-S186A-* GFP_3	
pMR6413	*CEN URA3* P_{GAL}*-cla4(281-330)-* GFP_3	Truncation-2
pMR6412	*CEN URA3* P_{GAL}*-cla4(281-330)-S308A-* GFP_3	
pMR6289	*CEN URA3* P_{GAL}*-cla4(315-364)-* GFP_3	Truncation-3
pMR6290	*CEN URA3* P_{GAL}*-cla4(315-364)-S351A-* GFP_3	
pMR6429	*CEN URA3* P_{GAL}*-cla4(381-424)-* GFP_3	Truncation-4
pMR6230	*CEN URA3* P_{GAL}*-cla4(381-424)-2A(S396A S398A)-* GFP_3	
pMR6428	*CEN URA3* P_{GAL}*-cla4(381-424)-4A(S396A S398A S400A S402A)-* GFP_3	
pMR6431	*CEN URA3* P_{GAL}*-cla4(381-424)-5A(S396A S398A S400A S402A S404A)-* GFP_3	
pMR6427	*CEN URA3* P_{GAL}*-cla4(406-460)-S445A-* GFP_3	Truncation-5

pMR6426	*CEN URA3 P_{GAL}-cla4(406-460)-S425A S445A-GFP$_3$*	
pMR6291	*CEN URA3 P_{GAL}-cla4(433-482)-GFP$_3$*	Truncation-6
pMR6292	*CEN URA3 P_{GAL}-cla4(433-482)-S445A-GFP$_3$*	
pMR6425	*CEN URA3 P_{GAL}-cla4(471-520)-GFP$_3$*	Truncation-7
pMR6424	*CEN URA3 P_{GAL}-cla4(471-520)-T492A-GFP$_3$*	
pMR6419	*CEN URA3 P_{GAL}-cla4(641-680)-GFP$_3$*	Truncation-8
pMR6417	*CEN URA3 P_{GAL}-cla4(641-680)-S663A-GFP$_3$*	
pMR6416	*CEN URA3 P_{GAL}-cla4(641-680)-S655A-GFP$_3$*	
pMR6418	*CEN URA3 P_{GAL}-cla4(641-680)-S663A S655A-GFP$_3$*	
pMR6423	*CEN URA3 P_{GAL}-cla4(722-800)-GFP$_3$*	Truncation-9
pMR6420	*CEN URA3 P_{GAL}-cla4(722-800)-T731A-GFP$_3$*	
pMR6421	*CEN URA3 P_{GAL}-cla4(722-800)-T785A-GFP$_3$*	
pMR6422	*CEN URA3 P_{GAL}-cla4(722-800)-T731A T785A-GFP$_3$*	

Yeast two-hybrid plasmids		Source*
pMR6995	*pEG202 for construction of LexA fusion protein, HIS3, 2µm*	5
pMR6996	*pJG4-5 for construction of B42AD fusion proteins, TRP1, 2µm*	5
pMR7002	*B42AD-CLA4 in pJG4-5*	
pMR7004	*B42AD-cla4(S186E) in pJG4-5*	
pMR7012	*LexA-cla4(C188S) in pEG202*	5
pMR7016	*B42AD-cla4-PBD (184-242) in pJG4-5*	
pMR7018	*B42AD-cla4-PBD-S186E (184-242) in pJG4-5*	

*All plasmids without a source indicated were constructed in this study.

1. Sikorski RS, Hieter P (1989) A system of shuttle vectors and yeast host strains designed for efficient manipulation of DNA in *Saccharomyces cerevisiae*. *Genetics* 122(1): 19-27.
2. Matheos D, Metodiev M, Muller E, Stone D, Rose MD (2004) Pheromone-induced polarization is dependent on the Fus3p MAPK acting through the formin Bni1p. *J Cell Biol* 165(1): 99-109.
3. Ydenberg CA, Rose MD (2009) Antagonistic regulation of Fus2p nuclear localization by pheromone signaling and the cell cycle. *J Cell Biol* 184(3): 409-422.
4. Versele M, Thorner J (2004) Septin collar formation in budding yeast requires GTP binding and direct phosphorylation by the PAK, Cla4. *J Cell Biol* 164(5): 701-715.
5. Heinrich M, Kohler T, Mosch HU (2007) Role of Cdc42-Cla4 interaction in the pheromone response of *Saccharomyces cerevisiae*. *Eukaryot Cell* 6(2): 317-327.
